# MyTaxa: an advanced taxonomic classifier for genomic and metagenomic sequences

**DOI:** 10.1093/nar/gku169

**Published:** 2014-03-03

**Authors:** Chengwei Luo, Luis M. Rodriguez-R, Konstantinos T. Konstantinidis

**Affiliations:** ^1^Centre for Bioinformatics and Computational Genomics, and School of Biology, Georgia Institute of Technology, Atlanta, GA 30332, USA and ^2^School of Civil and Environmental Engineering, Georgia Institute of Technology, Atlanta, GA 30332, USA

## Abstract

Determining the taxonomic affiliation of sequences assembled from metagenomes remains a major bottleneck that affects research across the fields of environmental, clinical and evolutionary microbiology. Here, we introduce MyTaxa, a homology-based bioinformatics framework to classify metagenomic and genomic sequences with unprecedented accuracy. The distinguishing aspect of MyTaxa is that it employs all genes present in an unknown sequence as classifiers, weighting each gene based on its (predetermined) classifying power at a given taxonomic level and frequency of horizontal gene transfer. MyTaxa also implements a novel classification scheme based on the genome-aggregate average amino acid identity concept to determine the degree of novelty of sequences representing uncharacterized taxa, i.e. whether they represent novel species, genera or phyla. Application of MyTaxa on *in silico* generated (mock) and real metagenomes of varied read length (100–2000 bp) revealed that it correctly classified at least 5% more sequences than any other tool. The analysis also showed that ∼10% of the assembled sequences from human gut metagenomes represent novel species with no sequenced representatives, several of which were highly abundant *in situ* such as members of the *Prevotella* genus. Thus, MyTaxa can find several important applications in microbial identification and diversity studies.

## INTRODUCTION

Culture-independent whole-genome shotgun (WGS) DNA sequencing has revolutionized the study of the diversity and ecology of microbial communities during the last decade (1,2). However, the tools to analyze metagenomic data are clearly lagging behind the developments in sequencing technologies, with the probable exception of tools for sequence annotation and assembly (1,3–5). Perhaps most importantly, the taxonomic identity of most sequences assembled from a metagenomic dataset frequently remains elusive, making the exchange of information about an organism or a DNA sequence challenging when a name for it is not available. This limitation severely impedes communication among scientists and scientific discovery across the fields of ecology, systematics, evolution, engineering and medicine. The limitation is due, at least in part, to the fact that the great majority of microbial species in nature, >99% of the total in some habitats (6), resist cultivation in the laboratory and thus, are not represented by sequenced reference representatives that can aid taxonomic identification. Single-cell techniques can potentially overcome these limitations by providing the genome sequence of uncultured organisms (7). However, these techniques are not amenable to all organisms or habitats and the 16S rRNA gene, which serves as the best marker for taxonomic identification due to the availability of a large database of 16S rRNA gene sequences from uncultured organisms (8,9), is often missed or not assembled during single-cell (and WGS metagenomic) approaches (10). The 16S rRNA gene also provides limited resolution at the species level, which represents a major limitation for epidemiological and micro-diversity studies (11). To overcome these limitations, whole-genome-based approaches and tools, comparable to those already available for the 16S rRNA gene, are highly needed. It is also important for these tools to scale with the increasingly large volume of sequence data produced by the new sequencers and to be able to detect and categorize novel taxa, e.g. determine if the taxa represent novel species or genera.

The previous methods to taxonomically identify metagenomic sequences fall into two categories: composition-based, such as PhyloPythiaS and NBC (12,13); and homology-based, such as CARMA3, SOrt-ITEMS, and MEGAN4 (5,14,15). While composition-based methods do not depend on the availability of a reference database for homology search (although most methods require a reference database for algorithm training purposes) and are typically faster to compute, their accuracy is usually significantly lower than homology-based methods, especially for regions of the genome that are characterized by abnormal statistics compared to the genome average, due, for instance, to horizontal gene transfer (HGT) (16). On the other hand, homology-based approaches such as those employing BLAST (17) and HMMER3 (18) searches of assembled or unassembled sequences against known reference database(s), have become a nearly indispensible component of metagenomic studies (4). Even naïve implementations of simple classification algorithms such as best hit (BH) or lowest common ancestor (LCA) usually provide comparable accuracies with some sophisticated composition-based approaches (19). The main limitation of the homology-based approaches is the lack of a comprehensive database of reference genome sequences. Accordingly, query sequences representing novel taxa provide only low-identity matches or no matches to the reference sequences and, in a typical metagenomic study, the majority of sequences cannot be robustly classified. Low-identity matches represent a challenge to the identification of the degree of novelty of the query sequence, particularly for naïve classifiers, which are based on pre-set, and frequently arbitrary, thresholds. In such cases, a dynamic approach that takes into account the level of identity of the match and the classification power of the corresponding gene or sequence (e.g. the 16S rRNA gene provides robust resolution at the genus level and higher but poor resolution at the species level) are advantageous. However, most, if not all, of the dynamic approaches developed for these purposes rely on some unrealistic assumptions such as that genes of the same protein family are characterized by the same mutation rate within different lineages (4,5,14).

Here we present a novel framework, MyTaxa, which overcomes several of the previous limitations and can accurately classify metagenomic and genomic sequences with low computational requirements. MyTaxa considers all genes present in an unknown (query) sequence as classifiers and quantifies the classifying power of each gene using predetermined weights. The weights are for (i) how well the gene in question resolves the classification at a given taxonomic level based on its degree of sequence conservation (e.g. 16S rRNA gene example above), and (ii) how frequently the gene phylogeny deviates from the species phylogeny due (primarily) to HGT. Based on these weights and the top homology matches of the genes in the query sequence against a pre-clustered reference gene database, a maximum likelihood analysis is performed to choose the most probable taxonomic assignment and to decide the lowest taxonomic rank for the query sequence. We show that MyTaxa significantly outperforms state-of-the-art tools for the same purposes in both sensitivity and specificity of the taxonomic assignments and can easily incorporate additional reference gene sequences as these become available through future isolate genome and single-cell sequencing projects to provide for a more comprehensive coverage.

## MATERIALS AND METHODS

### Overview of the MyTaxa algorithm and webserver

**Figure 1.**
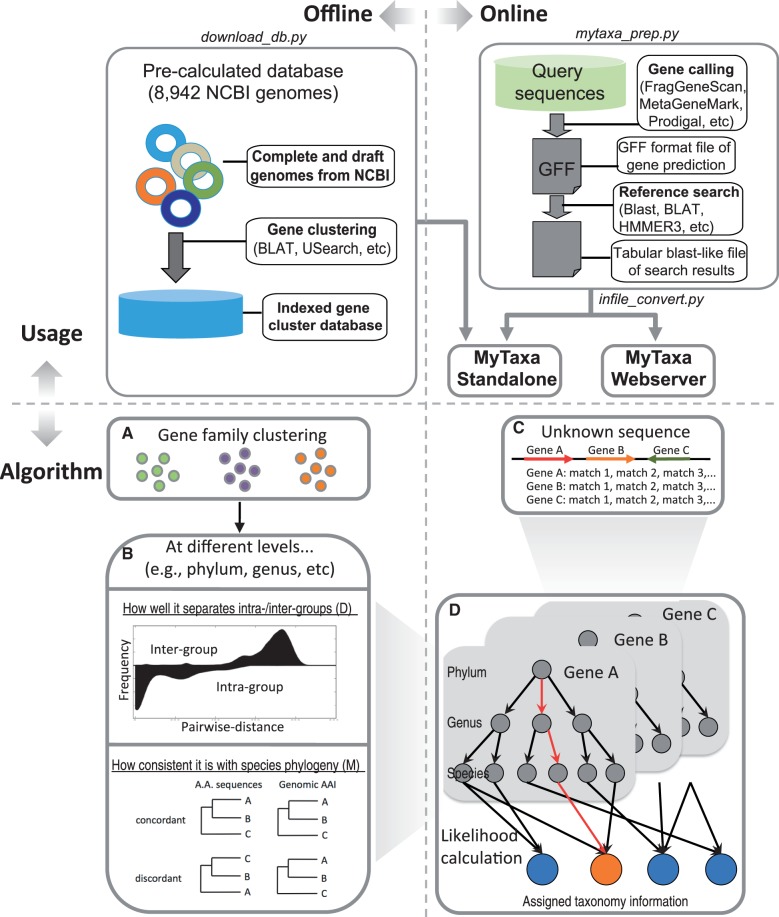
The workflow of the MyTaxa algorithm. (Top) Using MyTaxa involves two parts: (i) the construction of a database that contains the weights for each gene cluster (offline part). The database is provided as part of the standalone implementation package of the algorithm. (ii) The user supplies the query sequences and the results from a similarity search of the sequences against a database such as GenBank (online part). The user can use either the webserver or the standalone implementation of MyTaxa (right). (Bottom) In the offline part, all genes from available complete or draft genomes were grouped into clusters (box A), and the weights *D* (how well the gene resolves the taxonomic rank) and *M* (how consistent the gene phylogeny is to the species phylogeny) were calculated for each cluster and taxonomic rank considered (i.e. phylum, genus and species). To quantify *D*, the distances among all gene sequences of a gene cluster were calculated in a pair-wise mode and categorized into ‘intra-group’ (the two corresponding genomes that encode the genes were assigned to the same taxon) and ‘inter-group’ (the two genomes were assigned to different taxa). The larger the difference between the inter-group versus the intra-group identities, the larger the classifying power of the gene with respect to *D* (an example of the distribution of identities is represented by the histogram shown in box B). To quantify *M*, all possible triplets from the phylogenetic tree of all sequences of a gene cluster were extracted and compared with the species tree, the latter approximated by the AAI tree (distance tree). Therefore, the triplets were either ‘concordant’ or ‘discordant’ with species tree (lower panel in box B). During the sequence assignment (‘online’ part; Box C). MyTaxa takes the user input and maps the matches onto the reference gene clusters generated from the offline part, based on the accession numbers of the (matching) genes from GenBank. The corresponding *D* and *M* weights are extracted for each rank that the taxon encoding the matching gene sequence is assigned to. If different genes of a query sequence or matches of a single gene suggest different classifications (i.e. matching taxon differs), each classification receives a likelihood score by merging the identity of the match and the corresponding *D* and *M* weights. If the total likelihood score of a classification (from the sum of the likelihoods of each match that supports the exact same classification) is below a minimum threshold, the classification is discarded. MyTaxa reports the classification that receives the largest likelihood score above the threshold at each taxonomy rank, together with its likelihood score (marked in red in box D).

MyTaxa consists of two parts: the ‘offline’ and the ‘online’ part (Figure 1). The offline part refers to the construction of an indexed database that contains the parameters (weights) for gene clusters, which are employed in the online part for taxonomic classification. The indexed database is freely accessible for download and standalone implementations at MyTaxa’s website, using the utility script ‘download_db.py’. The database will be updated at regular intervals (twice a year). The current version is constructed using 8942 publicly available genomes in NCBI (release 196).

For the online part, users can use either the webserver (http://enve-omics.ce.gatech.edu/MyTaxa/) or the standal-one version. In the webserver, users can start MyTaxa analysis by supplying two files: (i) a standard GFF file containing the genes predicted on the query sequences by gene prediction tools such as metaGeneMark, Prodigal or FragGeneScan (20–22); and (ii) a tabular output file from the similarity search of the predicted gene sequences against the sequences used to construct the database of gene weights or another database that includes the GI accession number of the matching gene. The similarity search can be performed using any search tool that provides the sequence identity of the match such as Blast, BLAT or USearch (17,23,24). The stand-alone version uses the same input format as the webserver, and a utility script ‘infile_convert.py’ is provided to generate the appropriately formatted input file for MyTaxa form the output of the similarity search file. Alternatively, if users have only un-annotated assembled contig or genome query sequences, a Python pipeline, ‘MyTaxa_prep.py’, takes these sequences as input in a multi-fasta format and performs gene calling, similarity search and formatting to output files ready for MyTaxa analysis. The output from MyTaxa is an XML file that supports interactive visualization of query sequences composition by Krona (25) using utility script ‘MyTaxa.distribution.pl’; for webserver users, the Krona visualization is automatically generated (Figure 1). The following sections explain in detail the offline and online parts of MyTaxa, including the mathematical formulation, parameter selection and benchmarking.

### Gene clustering

The predicted protein-coding genes of 2453 completed and 6489 drafted microbial genomes were downloaded from NCBI’s FTP server (ftp.ncbi.nih.gov) in June 2013 (GenBank release 196). An all-versus-all search of all genes was carried out using USearch (version 5.0; global alignment algorithm with default settings) (23). Orthologs were defined as the reciprocal best match (RBM) genes between any two genomes, with percentage amino acid identity (AAI) >40%, no >70% coverage of the length of the shorter gene by the alignment, and *e*-value <1 × 10^−^^12^. Neo4j (www.neo4j.org) was subsequently used to construct a graph in which the nodes were genes and the edges were RBM relationships. Genes were grouped into gene clusters based on the graph by an agglomerative hierarchical clustering approach until all connected components of the graph were found. Non-RBM (paralog) genes were searched against the resulting gene clusters using the same USearch search as described above; genes with matches above the previous cut-off were merged into the corresponding best-match gene cluster. In total, 4 357 681 gene clusters (singletons included) comprising 29 911 689 genes were obtained. This approach was preferred over the clustering approach offered by the USearch software because it was computationally more efficient and provided, in addition, the data to calculate the AAI values among genomes (see below).

### Genome-aggregate average AAI

**Figure 2.**
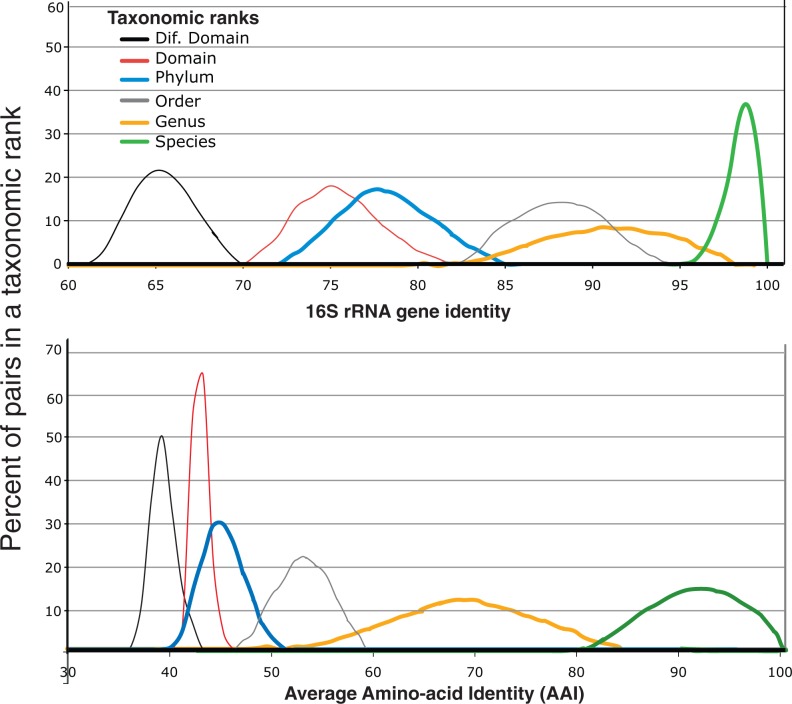
Relationships between taxonomic designations and genome-aggregate average AAI. The taxonomic designations of 410 fully sequenced genomes were compared to identify the lowest taxonomic rank shared by each pair of genomes [410 × (410 – 1) = 167 690 pairs, in total], essentially as described previously (27). For each taxonomic rank (figure key), the corresponding line shown represents the distribution of the 16S rRNA gene identity (top) and AAI (bottom) values among all genomes grouped at the rank.

To measure the overall genetic relatedness between any two genomes, we used the AAI, a robust and universal measure for these purposes (26). AAI was calculated as the arithmetic average of the AAI of all RBM conserved genes between two genomes. By comparing the AAI values among genome pairs grouped at different taxonomic ranks (e.g. phylum, class), it became evident that phyla, genera and species are clearly distinguishable from each other but that was not the case for the remaining taxonomic ranks, which overlapped extensively (Figure 2; see also Results section). Therefore, MyTaxa considers only these three taxonomic ranks when classifying query sequences that represent novel (uncharacterized) taxa; for sequences representing characterized taxa, all available taxonomic ranks for the matching taxon are provided in MyTaxa’s output.

### Gene cluster parameterization

We quantified the classifying power of each gene cluster at each of the three taxonomic ranks. The classifying power was defined by: (i) how well the gene separates intra-group members from inter-group ones based on the degree of sequence conservation (measured by *D*). For instance, the 16S rRNA gene is highly conserved and thus can resolve well the phylum and genus levels but poorly the species level; several rapidly evolving protein-coding genes resolve well the species and genus levels but poorly the phylum level (e.g. permissible mutations are saturated at the phylum level). And (ii) how consistent the gene phylogeny is with the species phylogeny, the latter approximated by the AAI distance tree (measured by *M*; Figure 1, box A).

To quantify *D*, the identities (or distances) among all gene sequences of a gene cluster were calculated in a pair-wise mode and categorized into ‘intra-group’ (the two corresponding genomes that encode the genes were assigned to the same taxon) and ‘inter-group’ (the two genomes were assigned to different taxa). The distributions of the distances of the two categories were then estimated by a kernel density estimator with a Gaussian kernel function using bandwidths selected by Scott’s rule (28). Therefore, the classifying power of a gene cluster at a specific sequence similarity level could be quantified as the difference between the inter-group and intra-group pair-wise distance distributions (Figure 1, box B).

To quantify *M*, first all possible triplets from all genes in a cluster were generated, and then for each triplet, a phylogenetic tree was constructed using FastTree (29) based on the MUSCLE (30) alignments of the gene sequences (default settings were used for both algorithms). Each triplet tree was compared against the corresponding species tree constructed based on AAI values. Therefore, the triplets were either ‘concordant’ (tree topology consistent with species tree), or ‘discordant’ (topology inconsistent with species tree). Hence, the degree of gene cluster *c* being consistent with the species phylogeny at taxonomic level *t* is defined as the percentage of concordant triplets among all possible triplets (Figure 1, box B; see Supplementary Material for the detailed mathematical formulations for *M* and *D*). For 40 gene clusters that had >5000 members, it was computationally prohibitive to exhaust all possible triplets among the members. We employed a Monte-Carlo method to estimate the *M* values for these gene families (see Supplementary Material for more details).

### Classification step and likelihood score calculation

For an unknown query sequence, MyTaxa gathers various pieces of information (i.e. matching genes and genomes, percentage identities, bit-scores of matches) from a similarity search of the gene sequences encoded on the query against a reference database. For practical reasons, only the top *N* matches of a gene are used (we recommend *N* = 5, see below). For every match of a gene encoded on the query sequence, MyTaxa calculates the likelihood that the query sequence could be assigned to the taxon that encodes the matching gene, using the bit-score of the match and the (pre-computed) weights for the gene. The weight ranges [0, 1], and it is a linear integration of *M* and *D* using a weight vector (details in the following section). Subsequently, the weights of all matches to genes of the same taxon are summed and the likelihood that the query sequence originated from that taxon is calculated as the percentage of the total weight for all matching taxa, i.e. (sum of weights for one taxon)/(total weights for all matching taxa). Note that if the query sequence encodes multiple genes and these match genes of the same taxon during the similarity search, the taxon is supported by multiple matches and hence, receives a higher fraction (and thus, likelihood) of the total weight of all matching genes (see Supplementary Material for more details).

If the top-scoring taxon at a given rank passes the likelihood score cut-off (see also score cut-off estimation below), MyTaxa predicts the query sequence to belong to this specific taxon and moves to the lower rank (if any) and calculates the likelihood score for this rank in a similar fashion. MyTaxa continues this procedure until either it reaches the lowest rank possible or the score falls below the threshold.

### Weight optimization based on a grid search

*D* and *M* weights were generated independently and thus, could not be integrated directly. To find the optimal combination of *D* and *M*, we defined (*w^D^*, *w^M^)* as the relative power of these two parameters, and the combined weight was 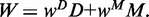 The sum of *w^D^ w^M^* should equal 1; therefore, we only need to optimize the algorithm performance over one of them, e.g. *w^D^*. A grid search was employed for this purpose and the 1000-bp query dataset was used. The dataset originated from available genomes (see below) and thus, its composition was known. For each possible (*w^D^*, *w^M^)* pair (*w^D^* was set to be 0.05, 0.1, 0.15, … , 0.95), we sampled 10% of the 1000-bp test dataset at random ten times (replicates) and make MyTaxa assignments. The assignments were evaluated by their accuracy (sensitivity and specificity analysis), and the corresponding weight pair with the highest accuracy was selected.

### Impact of the number of matches and score cutoffs on classification accuracy

In MyTaxa, the top *N* numbers of matches of genes are used in predicting the taxonomic identity of the query sequence. When *N* = 1, MyTaxa is equivalent to a weighted LCA algorithm; and when *N* = ∞, MyTaxa considers all matching taxa in the reference database. It follows that the larger the *N* value the larger the CPU and memory requirements. The choice of *N* has a complicated impact on prediction accuracy, and for practical reasons, we have tested *N* = 1, 2, … , 10, and found that for most cases, *N* = 5 offers optimal performance (Supplementary Figure S1). Similarly, we evaluated the impact of likelihood score cutoffs on accuracy. We found that 0.5 usually provides the best performance (Supplementary Figure S2).

### Comparisons against other tools

All comparisons against other classification tools were based on the following datasets. The *in silico* query datasets were composed of different combinations of 1687 draft microbial genomes, which were downloaded from NCBI’s ftp site in February 2012 (GenBank release 188). A custom Perl script was employed to randomly sample pieces of sequences from the drafted genomes at designated lengths (e.g. 100, 800 bp; see Supplementary Table S1) with simulated 1% sequencing error. The 1480 completed genomes available in GenBank release 188 were used as the reference database to calculate the offline parameters for MyTaxa, following the same procedure as described above. MyTaxa was subsequently applied on the *in silico* query datasets using default settings, i.e. score cutoff: 0.5 and number of hits to use: 5. Publicly available tools (NBC, MEGAN4, RAIPhy) were also trained on the same reference database as MyTaxa (1480 completed genomes) before applied to the (same) query datasets, using recommended or default settings. Note that MyTaxa analysis of real metagenomes was based on the indexed database derived from GenBank release 196, which was 10.9% larger than release 188. Also note that the PhyloPythiaS webserver requires, by default, at least three genomes available in a taxon for model construction. It was trained based on GenBank release 184 (1332 genomes), and it is not recommended for sequences <1000 bp. For MG-RAST, it was not possible to train the underlying algorithm on our reference database. Therefore, the results of the latter two tools may not be directly comparable to those of MyTaxa and other tools.

The sequences in the query datasets were labelled ‘known’ or ‘unknown’ depending on whether or not a homologous sequence from the same species was available in the reference database. The taxonomic assignment of a ‘known’ sequence by MyTaxa or another tool was denoted as ‘true prediction’ (TP; predicted taxon matches the actual taxon), ‘wrong prediction’ (WP; predicted taxon does not match the actual taxon) or ‘false negative’ (FN; predicted as ‘unknown’); while the assignment of an ‘unknown’ sequence was denoted either ‘false positive’ (FP; predicted to match a specific taxon) or ‘true negative’ (TN; predicted as ‘unknown’). Accordingly, the sensitivity (i.e. normalized fraction of total sequences that were TP or TN) and the specificity (i.e. normalized fraction of total ‘known’ sequences that were TP) of the algorithm at a given rank was defined in a categorized fashion, which essentially is a weighted performance of the algorithm over all taxa on that rank, including known and unknown (see Supplementary Material for more details).

### Degree of novelty of the query dataset

To test the impact of the degree of novelty of the query dataset (defined as the percentage of query sequences originated from taxa that are ‘unknown’ to reference dataset) on the performance of MyTaxa, the following approach was employed. The 8942 available genomes from GenBank release 196 were categorized into low, medium and high abundance based on the number of genomes available for the species, genus and phylum that the genomes are assigned to according to NCBI’s taxonomy (see Supplementary Figure S7 for details on what number of genomes was used as threshold for each taxonomic rank). These genomes were sampled at random, as described above for the *in silico* query metagenomes, keeping the relative abundance of sequences from each of the three categories roughly the same to ensure a fair comparison among the three categories (Supplementary Table S4). For each resulting query dataset, a custom reference database was generated using all genomes (complete and draft) but those used to construct the query dataset. We removed increasingly more genomes and used the remaining genomes for parameter training so that the novelty of the query dataset relative to the reference database ranged from 2 to 54% for each of the three different taxonomic ranks evaluated (Supplementary Table S4).

### Analysis of real metagenomes

The human stool data were downloaded from the HMP Consortium webpage (www.hmpdacc.org; SRA accession numbers SRS011405, SRS011529 and SRS011586). The biogas-producing metagenome was downloaded from the FTP site as documented in (31). The gene sequences on the scaffolds were predicted using FragGeneScan (22) with default settings, and were subsequently searched against all genomes in NCBI using BLAT (23). The resulting data were used for taxonomic assignment of the scaffolds by MyTaxa, based on default settings. Trimmed paired-end reads were mapped onto the scaffold to calculate the coverage (*in situ* abundance) of the corresponding population/genotype using BLAT with default settings and a minimum cut-off for a match of 50 bp aligned length, 97% nucleotide identity and 1*e* – 10 *e*-value. Taxon abundance was estimated using the percentage of total reads mapping on all scaffolds assigned to the taxon, normalized to the average genome size of the taxon reported in the literature. For comparison (e.g. Figure 6), the relative abundance of reference genomes based on the number of paired-end reads mapping to available genome sequences were directly obtained from the HMP consortium webpage (www.hmpdacc.org/HMSCP).

## RESULTS

### Standardizing novel taxa based on average AAI

For reliable and high-throughput taxonomic classification of unknown sequences, it is essential to have a robust and standardized reference taxonomy system. The current taxonomic system, especially the ranks higher than the species rank, is primarily based on the grouping patterns of the 16S rRNA gene phylogeny but no standards exist on the degree of genetic relatedness of the organisms grouped at different ranks. Accordingly, adjacent ranks are highly overlapping with this respect. For instance, organisms representing different species of the same genus are often (30% of the cases examined, on average) as divergent from each other as many genera of the same family are (32). These inconsistencies can complicate taxonomic identification of unknown sequences. Indeed, several commonly used approaches including PhyloPythiaS and MEGAN have significantly lower specificity above the species level (13).

To examine in depth the inconsistencies in the current classification system, we analyzed 400 closed bacterial genomes from which we exhausted all possible genome pairs using the AAI to measure the genetic relatedness among the genomes (26). Our results confirmed previous findings (32) that high overlap exists not only among adjacent ranks (e.g. phylum versus domain), but also revealed that the species, genus and phylum ranks are rarely overlapping, i.e. the inter-taxon divergence is typically higher than the intra-taxon diversity for these three ranks (Figure 2). In particular, organisms grouped at the ‘species’ level typically show >85% AAI among themselves and are clearly distinguishable from those grouped at the genus (showing 60–80% AAI) and the phylum levels (showing <45% AAI). MyTaxa essentially employs these AAI standards and examines the degree to which an individual gene reflects the genomic AAI (see Materials and methods section for details), to determine the taxonomic rank of a sequence representing a novel organism, i.e*.* a species, genus or phylum, with the following adjustment. For novel species, a cut-off of 95% AAI (instead of 85% AAI from Figure 2) was used because this was found to better represent recently described species, which are, in general, more homogenous than older species designations included in our analysis (33). Also note that the 45% AAI cut-off encompasses deep-branching organisms that a (future) detailed taxonomic analysis may in fact assign to (new) deep branching classes or even domains as opposed to phyla; we refer to all these cases as phylum-level lineages, or just phyla, for simplicity. Species, genera and phyla also represent the three most important ranks of prokaryotic taxonomy. Therefore, MyTaxa does not consider the remaining ranks of the taxonomy (family, order, etc.) when classifying novel taxa but these ranks are available for organisms assigned to known species, genera or phyla.

### Computing the weights of the classifying power of each gene

To determine the weights of each gene, we built clusters for all genes present in all completed and draft bacterial and archaeal genomes as of June 2013 (release 196; *n* = 8942). We determined the classifying power of each gene cluster by comparing how well the identity between two genes of the cluster reflected the taxonomic rank of the genomes encoding the genes, separately for each of the three taxonomic ranks considered. The idea is analogous to the use of AAI above to examine overlap between the taxonomic ranks (e.g. Figure 2), applied to individual genes. A second weight was calculated for each gene cluster based on how frequently the ortholog gene phylogeny deviates from the species phylogeny, the latter approximated by the AAI-based tree, due (primarily) to HGT (Figure 1). The weights were stored in a structured database, and the preceding analysis is referred to as the ‘offline’ part of MyTaxa (external users do not have to repeat this part). For the ‘online’ part, an external user submits a file that contains the results of a search, by BLAST, USearch or other algorithm, of the query sequence against the reference database of gene clusters. In fact, the search is not necessary to be against MyTaxa’s reference database as long as the input file contains the accession number of the best matching gene(s) in GenBank database and the AAI of the match. MyTaxa then employs a maximum likelihood analysis of the pre-calculated weights for the gene cluster that provided the best match of the query sequence and the identity of the best match to determine the taxonomic identity of the query sequence and provide a statistical probability for the assignment (Figure 1). Therefore, the most computationally intensive part, i.e. to update the weights of genes, is calculated offline once or twice a year, depending on the number of new genomes sequenced; and MyTaxa requires significantly lower computational resources during the online part of the analysis, comparable to other homology-based methods (see also below). For instance, the online part of MyTaxa can be run on a personal laptop with input sequences in the order of hundreds of megabyte to gigabyte in size.

### MyTaxa’s performance

We evaluated the performance of MyTaxa against that of other existing tools based on the following approach. For classifying sequences that represent organisms present in the database (100% AAI match) or close relatives of these organisms (e.g. >95% AAI for organisms of the same species), the algorithm should (correctly) identify the sequence to the lowest level available. The latter was typically the species level, unless the reference organism has not been classified at the species level yet. For sequences representing, for instance, an unknown (novel) genus of a known phylum, the algorithm should ideally identify the correct phylum, predict the phylum as the lowest taxonomic rank and denote it as a novel genus; similarly for novel phyla and species. Based on this framework, we compared the performance of MyTaxa with other available tools on both *in silico* generated (mock) and well-characterized, real metagenomes.

To prepare *in silico* generated test datasets, 1687 available draft genomes were sampled, at random, to produce six test datasets, each composed of sequences of different length, ranging from 100 (simulating Illumina reads) to 500 (simulating Roche 454 Titanium FLX reads), 800 (simulating Roche 454 FLX+ reads), 1000 (representing the average bacterial gene length), 1500 and 2000 bp (for details see Supplementary Table S2 and Figure S3). These sequences also had ∼1% (artificially introduced) sequencing error, which simulated the error rate and types observed in the Illumina GA-II and Hi-Seq 2000 sequencers (34). From GenBank, 1480 completed genomes served as the reference/training database to build the gene clusters and associated weights for MyTaxa and other tools, when appropriate (Supplementary Tables S1 and S2). Thus, the sequences in the test datasets were labelled ‘known’ or ‘unknown’ depending on whether or not a homologous sequence of the same species as the draft genome was available in the reference complete genomes and the algorithms were evaluated on the number of correct assignments (predictions) made.

For the homology-based algorithms, we ran a BLAT (23) search of the gene sequences predicted on the six query datasets by FragGeneScan (22) against the reference database, and the search results were used as input for the algorithms. For composition-based algorithms, the algorithms were trained, when appropriate, on the same reference database described above and then applied on the query dataset using default settings (see Materials and methods section for details). To achieve a fair comparison in terms of prediction accuracy given that composition-based methods tend to classify more sequences compared to homology-based methods (e.g. they do not depend on the availability of a match of the query sequence against a reference database), we split the input query sequences into two categories: the first one included queries with at least one significant match in the BLAT search (category A); the remaining queries formed the second category (category B). All methods were directly compared against each other based on the first category while the accuracy of the composition-based methods on the second category was evaluated separately. The percentage of query sequences assigned to the first category ranged from 32.7 to 51.2% of the total test dataset, depending on the dataset considered (Supplementary Table S2).

**Figure 3.**
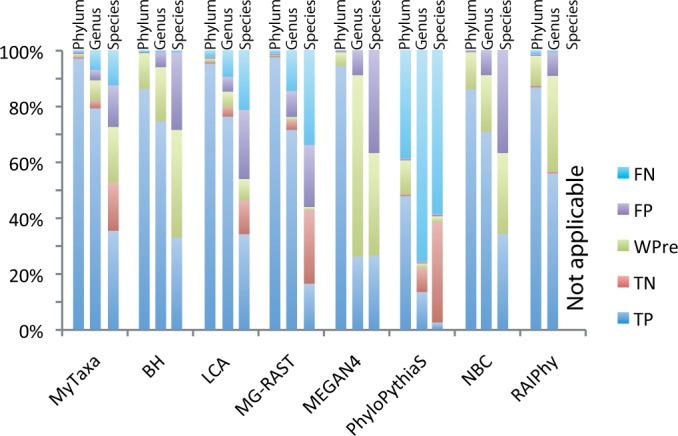
MyTaxa’s performance and comparison with other methods. Each bar represents the relative distribution of the different types of predictions (figure key) made by each of the methods evaluated (*x*-axis), at each taxonomic rank considered (labels on top). FN, false negative (sequence from a known taxon predicted as unknown); FP, false positive (sequence from an unknown taxon predicted as known); WPre, wrong prediction (the known taxon did not match the predicted taxon); TN, true negative (sequences from an unknown taxon were predicted as unknown); TP, true prediction (the known taxon matched the predicted taxon). The results are based on sequences of the 800-bp-long query test dataset that found a match against the reference database during the similarity search step (for the performance of composition-based methods on the remaining sequences of the dataset see Discussion section). Note that PhyloPythiaS (13) is not recommended for query sequences <1 Kb and taxa with less than three reference genomes available, and RAIPhy does not provide species level prediction; thus, the results of these two tools may not be directly comparable to those of MyTaxa and other tools.

The results revealed that MyTaxa consistently outperformed other homology-based tools (Supplementary Table S3 for all results; Figure 3 shows the results for the 800-bp dataset as a representative example; Wilcoxon rank test, *P* < 0.05). For instance, at the species level (800 bp test dataset), it was, on average, 17.8, 6.9, 25.1 and 9.2% more accurate, i.e. more correct predictions and/or fewer false predictions, than BH, LCA, MEGAN4 and MG-RAST, respectively. Furthermore, as the length of query sequences increased, the advantage of MyTaxa was more pronounced (Supplementary Figure S4). NBC provided more correct classifications compared to the other composition-based methods, consistent with previous findings (12). MyTaxa outperformed NBC by 10.6, 11.2 and 17.0% at the phylum, genus and species levels, respectively (average of category A sequences from all test datasets).

**Figure 4.**
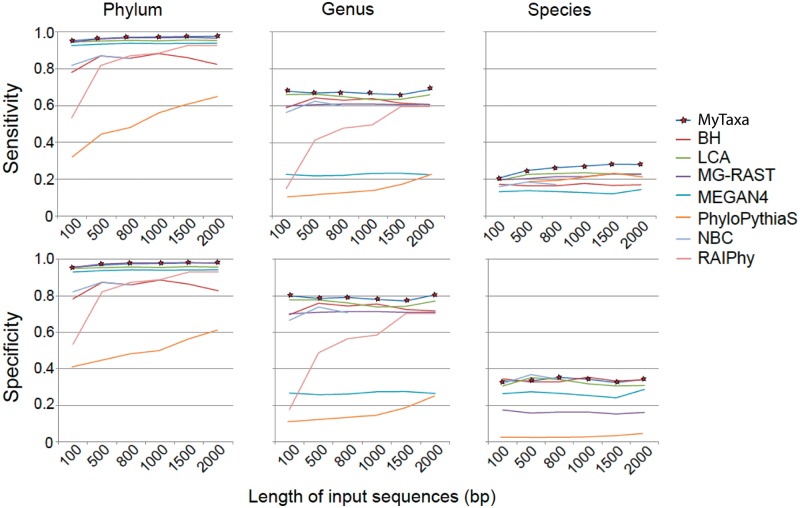
Sensitivity and specificity of MyTaxa and comparison with other methods. Each line represents the sensitivity (*y*-axes; upper panels) or specificity (*y*-axes; lower panels) of a tool (figure key) on different lengths of input sequences (*x-*axes). Sensitivity and specificity were defined as described in the text.

We also calculated the sensitivity (Sn; defined as the normalized portion of sequences from known taxa correctly assigned as known and the portion of sequences from unknown taxa corrected assigned as unknown) and specificity (Sp; defined as the portion of sequences from known taxa correctly predicted at the lowest rank possible) for all methods (Figure 4; see Supplementary Material for the mathematical formulations of Sn and Sp). MyTaxa showed both high sensitivity and specificity in all three taxonomic ranks evaluated, e.g. at species level it was on average 5% more sensitive and 3% more specific than any other tool. Moreover, the sensitivity and specificity of MyTaxa did not seem to depend on the length of the input sequences, while most composition-based approaches showed strong length-dependent variance in both sensitivity and specificity (Figure 4). Specificity and sensitivity combined, the improvement provided by MyTaxa, e.g. at least 3% more sequences correctly classified, was consistent with the results shown in Figure 3 and represents substantial difference compared to current practice for the field of taxonomic sequence assignment (35).

The run-time of the homology-based algorithms was also compared on a 2.4-GHz single CPU Linux node with 8-GB RAM using the 1000-bp dataset. The time required for the similarity search step was not taken into account, since it was the same for all tools (representative examples of the search time expected using different algorithms are listed in Supplementary Table S5), and the evaluation was limited to the time required to analyze the processed results (‘ready-to-go’ input files). Composition-based methods were not compared with this respect since they are based on a different algorithm strategy that does not include a similarity search step. Overall, the comparison showed that the online part of MyTaxa was as fast as, if not faster than, other homology-based tools (Supplementary Figure S6); for instance, it ran about two times faster than MEGAN4 (MEGAN4 settings were set to carry out taxonomic assignment only, not functional annotation of genes, in order for the search time to be directly comparable to that of MyTaxa).

**Figure 5.**
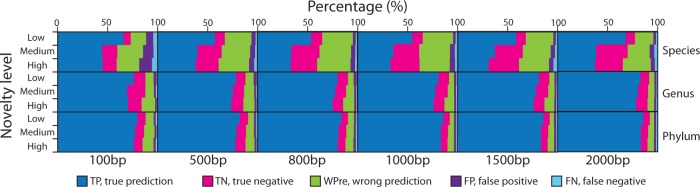
Evaluation of MyTaxa’s performance in query datasets of varied degrees of novelty relative to the reference database. Query datasets of varied degrees of novelty (low, medium and high; *y*-axes), at each taxonomic rank evaluated (species, genus and phylum; rows) and length of input query sequences (100, 500 bp, etc.; columns) were created from genome sequences available at NCBI. For each dataset evaluated, MyTaxa’s performance is represented by three stacked bars, one for each degree of novelty, that show the relative percentage of different prediction categories, specified and color-coded as in Figure 3. Note that the performance decreased as the novelty of the query datasets increased; however, the decrease was minor (<5% query sequences affected) for query sequences >500 bp, at all three taxonomic ranks.

To evaluate the impact of the degree of novelty of the query dataset relative to the reference database on the results obtained, i.e. the relative frequency of unknown vs. known taxa, a series of *in silico* query datasets, with novelty ranging from 2 to 54%, were constructed (for details, see Supplementary Table S4 and Materials and methods section). Not surprisingly, the degree of novelty negatively impacted MyTaxa’s prediction accuracy (Kendall’s tau test, *P* < 0.01). However, the impact was rather minor, affecting ∼5% of the total query sequences or fewer, for 500 bp sequences or longer, at all three taxonomic ranks evaluated (Figure 5 and Supplementary Figure S8). Other homology-based approaches (MEGAN4, BH and LCA) showed larger decreases in accuracy compared to MyTaxa on the same datasets (Supplementary Figure S8). These observations suggested that MyTaxa is a robust classifier.

For a real metagenome, the previously characterized biogas reactor dataset (31), which consisted of 616 072 Roche 454 FLX reads (read length: 230.0 ± 55.4 bp), was used. MyTaxa, NBC, MEGAN4, MG-RAST and RAIPhy were compared against each other based on their classification results for each read of the metagenome, while PhyloPythiaS was omitted from the comparison due to the low number of reads classified at the genus level (<5% of the total reads). MEGAN4 showed the highest dissimilarity compared to the results of the other methods based on the (predicted) relative abundance of the 21 most abundant genera in the dataset (Supplementary Figure S9). MyTaxa showed overall high similarity with NBC and MG-RAST. However, several notable differences in accuracy between the methods were also observed and were consistent with the results from the *in silico* generated datasets. For example, the genus *Alkaliphilus* was predicted to be relatively abundant by MyTaxa (2.23% of total reads) but not by NBC (0.34%) while *Pseudomonas* was predicted to be very abundant by NBC (5.47%), but not by MyTaxa (0.31%). Previous analysis based on manual inspection of the coverage of large contigs assembled from these reads revealed that the sample indeed contained >1% *Alkaliphilus metalliredigens* and <1% *Pseudomonas* (31), consistent with MyTaxa’s results.

### Novel diversity revealed in the human microbiome

We also evaluated MyTaxa’s ability to identify novel taxa using several metagenomes that were made available as part of the Human Microbiome Project (HMP). HMP data were chosen for this purpose because many isolate genomes have been obtained from the same samples; hence, the novel taxa in these samples should be comparatively fewer compared to other samples and more challenging to detect. The analysis was restricted to assembled scaffolds (not unassembled reads), which provide more reliable results in terms of novel taxa detection due to the longer available sequences. Note, however, that taxon relative abundance based on scaffolds may not necessarily match that based on unassembled data in cases where coverage is not uniform across the scaffold sequence length and/or a reliable estimation of the genome size of the target taxon/population is not available (36). For simplicity, the results from three representative stool HMP metagenomes, in terms of size and complexity, which included assembled scaffolds, are reported here. Each scaffold was classified by MyTaxa with default settings, and the relative abundance of the corresponding taxon was estimated based on the average number of reads mapping (coverage) on all scaffolds assigned to the taxon, essentially as described previously (36). The resulting taxon abundance profiles were also compared to the profiles available from the HMP website based on paired-end-read mapping to reference genomes (www.hmpdacc.org/HMSCP).

**Figure 6.**
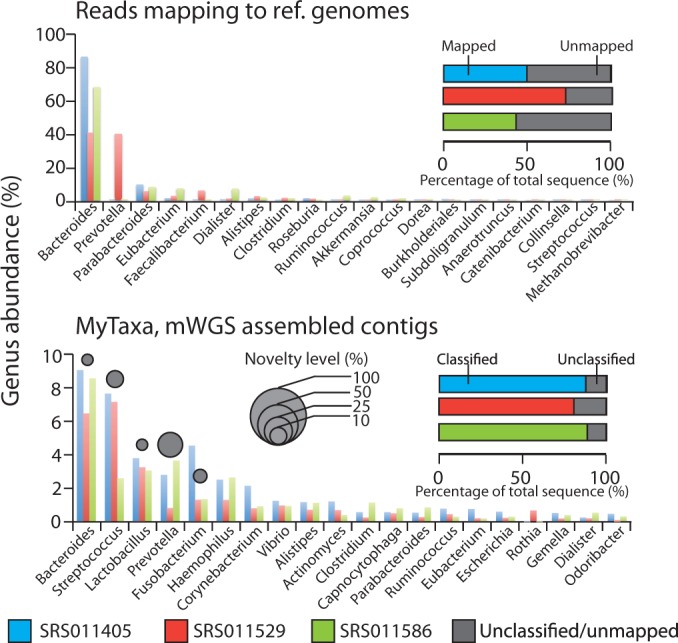
Genus-level community composition and abundance of novel taxa in the human microbiome based on MyTaxa and reference genome-mapping analysis. (Upper) Relative abundances of genera were obtained from the number of reads mapping to reference genomes; these data were downloaded from the HMP webpage. (Bottom) MyTaxa analysis based on assembled scaffolds, followed by mapping of metagenomic reads against the scaffolds using BLAT (24) to estimate relative abundance. The height of the bars represents the relative abundance of genera in each sample (*y-*axis), and the top twenty most abundant genera (*x-*axis) according to each method are shown. The percentage of classified or mapped sequences in each sample is represented by the horizontal bars. For MyTaxa, the top five most abundant genera were further analyzed for the degree of novel species they encompassed, defined as the percentage of sequences assigned to each genus that were not classifiable at the species level (grey circles, averages of the three samples are shown).

About half of the reads in each HMP metagenome did not map to any reference genomes, which included 131 archaeal, 326 eukaryotic and 1751 bacterial genomes originating from human samples, indicating that a large fraction of the corresponding communities is still not well represented by isolate genomes. The latter accounted, at least in part, for the differences observed between the taxonomic profiles of the samples based on MyTaxa (Figure 6A) relative to those based on reads mapping to reference genomes (Figure 6B). For instance, although both methods identified *Bacteroides* as the most abundant genus, MyTaxa found several abundant genera to be represented by the assembled contigs that were absent in mapping-based profiles. MyTaxa’s results showed that an average of 8.7% of the total assembled sequences assigned to each of the top 10 most abundant genera were contributed by novel species, which are not currently represented by genome sequences, draft or complete. Especially in *Prevotella* genus, representatives of which represent keystone members of the gut microbiome (2), novel species represented 24.3% of the total sequences assigned to this genus (Figure 6). To confirm the latter findings, all reads of the metagenome were mapped against available reference complete or draft *Prevotella* genomes. This analysis revealed that about half of the reads showed (only) 80–90% nucleotide identity to their best-matching reference genome (Supplementary Figure S10), indicating that they indeed represented novel species within the *Prevotella* genus (32).

## DISCUSSION

We have shown that MyTaxa accurately classifies at least 3%, and up to 25%, more query sequences compared to other methods for sequences representing previously described taxa, independent of the length of the sequences (Figure 3 and 4 and Supplementary Figure S4 and S5). The advantage of MyTaxa is rooted in the construction of a likelihood framework that integrates the matches of individual genes with weights for the classifying power of each gene to achieve a better prediction. This approach is categorized into a broad genre of optimizations, often referred to as ‘the wisdom of the crowd’. Indeed, a greater advantage of MyTaxa over other methods was observed when the query sequences were longer (Supplementary Figure S4), presumably due to more information (genes) available. Accordingly, MyTaxa can also facilitate taxonomic studies of whole genomes, complete or draft and be complementary to 16S rRNA gene-based classifications since it provides higher resolution at the species level. MyTaxa has also a clear advantage over other methods in identifying the rank of sequences representing novel taxa due to the use of an AAI-based framework that emerges from the current classification system but it is more standardized (Figure 2). This is particularly useful to the study of communities that are not well represented by reference genome sequences (the majority of microbial communities) and can help identify abundant, and thus, presumably important, members of the community that should be targeted for single-cell or cultivation efforts. Indeed, MyTaxa analysis of human microbiome samples revealed several abundant (novel) species that are not represented by genomes of isolates, despite the large number of isolates sequenced as part of the HMP (2208 genomes used in this article). For instance, although a large number of *Prevotella* isolate genomes are available (50 genomes, including draft genomes), MyTaxa suggested that several key members of this genus are still awaiting genomic characterization.

Despite the significant improvements achieved by MyTaxa, assigning sequences at the species level remains problematic; mostly due to the lack of representative sequences for several species (e.g. Figure 4). However, the recent developments in DNA-sequencing technologies, especially single-cell approaches, has greatly increased the number and phylogenetic diversity of available genomes so that the reference genome database will not represent such a major limitation in the near future. What, however, will still represent a limitation for automatic, high-throughput taxonomic identification are the inconsistencies in the current classification system. While we employed a standardized AAI-based system to determine the degree of novelty of a sequencing representing a novel taxon, we relied on the existing system for sequences representing previously described taxa. The weights of gene clusters are expected to significantly improve if a standardized system, which will limit overlap between adjacent taxonomic ranks in terms of the genetic relatedness of the grouped organisms, will become available for previously described taxa. MyTaxa is also scalable to a higher volume of input data in that the computational demand for the online part of the algorithm represents a linear function of the number of input sequences. MyTaxa is not specific to the homology search algorithm used; thus, if new faster algorithms become available, e.g. BLAT (24), they can be easily compatible with MyTaxa.

One advantage of composition-based methods is that they typically classify all input sequences, including those that show no significant homology to the reference database. However, the exact error rate of these predictions remains unclear, although it is generally assumed to be lower relative to that for sequences with significant matches. Genes with no significant homology to known genes are highly likely to represent taxon-specific functions (and thus, are not useful for classification if related genomes have not been already characterized) and their evolutionary history is often inconsistent with that of the genome (e.g. acquired via HGT) (37). For instance, in our datasets, NBC’s accuracy on ‘unknown’ sequences was on average 25% lower compared to those with matches in the reference database, which was largely attributable to a higher wrong prediction rate (Supplementary Figure S5). Therefore, if classifying more sequences is more important than high accuracy in the classifications, a hybrid approach that combines composition- and homology-based methods may be advantageous. Finally, a new homology-based tool to profile microbial communities was recently released, mOTU (38), which is essentially based on AAI values of a small set of 40 universal genes (as opposed to any gene in the genome used by MyTaxa). Preliminary data showed that mOTU and MyTaxa provide, in general, similar results for abundant taxa in available HMP metagenomes, albeit MyTaxa is able to detect a larger number of low abundance taxa, which are poorly represented among the sequences of the 40 universal genes assembled from the metagenomes (but not those of other genes in the genome), and provide a more accurate assessment of the level of novelty of sequences representing uncharacterized taxa due to its standardized AAI-based classification system.

In addition to the applications mentioned above, MyTaxa can also be used to assist studies that aim to detect HGT between genomes or contigs assembled from a metagenome and the genomes represented in the reference database, e.g. by scanning the query sequence in windows of specific length and compare the taxonomic affiliations of the resulting sequence fragments. It can also assist in validating the taxonomic identity of contigs binned into population genomes during metagenomic studies, especially for populations related to previously described taxa. Thus, MyTaxa can find several important applications in microbial identification and diversity studies and provide new insights into the highly complex microbial communities.

## SUPPLEMENTARY DATA

Supplementary Data are available at NAR Online.

## FUNDING

Funding for open access charge: US DOE Office of Science, Biological and Environmental Research Division (BER) (Award No. DE-SC0004601) (in part); US National Science Foundation (Award No 1241046) (in part).

*Conflict of interest statement*. None declared.

## Supplementary Material

Supplementary Data
