# Supplementary material for: MyTaxa: an advanced taxonomic classifier for genomic and metagenomic sequences
Source: Nucleic Acids Res. 2014 Mar 3;42(8):e73. doi: 10.1093/nar/gku169 (PMC4005636; doi:10.1093/nar/gku169)
Supplement: Supplementary Data [file supp_42_8_e73__index.html]

MyTaxa: an advanced taxonomic classifier for genomic and metagenomic sequences — Supplementary Data 

# MyTaxa: an advanced taxonomic classifier for genomic and metagenomic sequences

## Supplementary Data

files

**Files in this Data Supplement:**

- Supplementary Data - docx file
