# Supplementary material for: MyTaxa: an advanced taxonomic classifier for genomic and metagenomic sequences
Source: Nucleic Acids Res. 2014 Mar 3;42(8):e73. doi: 10.1093/nar/gku169 (PMC4005636; doi:10.1093/nar/gku169)
Supplement: Supplementary Data [file supp_gku169_Luo_et_al_NAR_SI.docx]

**Supplementary Information**

**MyTaxa: an advanced taxonomic classifier for genomic and metagenomic sequences**

Chengwei Luo, Luis M. Rodriguez-R, and Konstantinos T. Konstantinidis

Supplementary material includes:

**Supplementary methods**

1. Deriving weights of gene clusters.
2. Large gene cluster weight estimation using the Monte-Carlo approach.
3. Calculation of classification likelihood.
4. Mathematical definitions of sensitivity and specificity.

**Supplementary tables**

1. **Table S1.** The number of known and unknown taxa among draft genomes (*in-silico* query datasets) compared to completed genomes (reference database).
2. **Table S2.** The number of known and unknown sequences at different taxonomic ranks in the *in-silico* query datasets used for performance evaluation.
3. **Table S3.** Detailed performance of classification tools on *in-silico* query datasets.
4. **Table S4.** The datasets used for evaluating the impact of the degree of novelty of the query sequence on MyTaxa’s prediction accuracy.
5. **Table S5.** Database search time using different search algorithms.

**Supplementary figures**

1. **Figure S1.** The impact of the number of matches used in the analysis on the classification accuracy of MyTaxa.
2. **Figure S2.** The impact of the likelihood score cutoff on the classification accuracy of MyTaxa.
3. **Figure S3.** Composition of *in-silico* generated datasets at phylum level.
4. **Figure S4.** Accuracy of MyTaxa in comparison to other homology-based methods at the species level.
5. **Figure S5.** Performance of composition-based methods on sequences that did not have significant matches in reference database.
6. **Figure S6.** Run-time analysis of MyTaxa in comparison with other classification tools.
7. **Figure S7.** Classifying of taxa into low, medium, and high abundance based on the number of genomes used in the reference database.
8. **Figure S8.** Prediction accuracy of different tools as an effect of the degree of novelty of the input dataset for various read lengths.
9. **Figure S9.** Genera relative abundance in a biogas-producing metagenome based on different taxonomic classifiers.
10. **Figure S10.** Population structure of *Prevotella* in the HMP metagenome

**Supplementary methods**

**1. Deriving weights of gene clusters**

The classifying power, *D*, of a gene cluster *c* as a function of the amino acid identity of the gene obtained from the gene sequence comparison (*e.g.*, a query sequence against the database) *h,* denoted as $D_{t}^{c}(h)$, for a given taxonomic rank *t*, was calculated as:

$D_{t}^{c}(h)=\frac{\int_{h}^{1} f_{t}^{c}(s)ds}{\int_{h}^{1} f_{t}^{c}(s)ds+\int_{h}^{1} g_{t}^{c}(s)ds}$ ,

where $f_{t}^{c}(s)$and $g_{t}^{c}(s)$ denote the distributions of the intra- and inter-group distances for gene cluster *c* at taxonomic level *t*, respectively (Fig. 2, box B).

The second classifying power, *M*, for gene cluster *c* being consistent with the species phylogeny at taxonomic level *t*, denoted as $M_{t}^{c}$, was calculated as:

$M_{t}^{c}=\frac{N_{t}^{c}(concordant)}{N_{t}^{c}(concordant)+N_{t}^{c}(discordant)}$ ,

where $N_{t}^{c}(concordant)$ denotes the number of concordant triplets and $N_{t}^{c}(discordant)$ denotes the number of discordant triplets for gene cluster *c* at taxonomic level *t* (Fig. 2, box B).

**2.** **Large gene cluster weight estimation using the Monte-Carlo approach**

For gene clusters with more than 5,000 members (40 such clusters were obtained, in total), it was computationally prohibitive to exhaust all possible triplets among the members to estimate classifying power weight, *M*. A Monte-Carlo method was employed to estimate the *M* values for these gene families. The method was applied as follows, separately for each of the three taxonomic ranks considered:

1. Initialization;

Set *M*=*s*, *N=*number of genes in the cluster; set number for concordant triplet, *c=*0; and number of discordant triplet, *d*=0.

1. Random sampling;

10,000 gene triplets are sampled from the gene cluster uniformly at random without replacing; the corresponding species triplets are constructed from the species tree.

1. Calculate new *M*;

The 10,000 gene triplets are compared against the corresponding species triplets. If they are concordant, then *c*=*c+*1; otherwise *d*=*d+*1. The new *M*, *M’*, is calculated as *M*’=*c/(c+d)*.

1. Termination.

If |*M’-M*|<0.01, then exit the algorithm and return *M*’;

Otherwise, *M*=*M*’, return to step 2.

To avoid being trapped at local maxima, we repeat the process with initializing *M* to be different values (in 0 to 1 with 0.1 increment, *e.g.*, 0, 0.1, 0.2, …, 1.0). If there were multiple estimated *M*’s, we repeat the process until it converges to a single value.

1. **Calculation of classification likelihood**

For an unknown query sequence *U* (*e.g.*, an assemble contig from a metagenome), we denote the genes encoded on it as $G=\{g_{1},g_{2},...,g_{n}\}$. These genes are searched against a reference database (*e.g.*, MyTaxa’s reference database~~gene clusters described above~~) and the returned *m* matching genes for *g_i_* are denoted as $H_{i}=\{h_{1},h_{2},...,h_{m}\}$, the corresponding percentage amino acid identities as $I_{i}=\{i_{1},i_{2},...,i_{m}\}$, and the bit-scores as $S_{i}=\{s_{1},s_{2},...,s_{m}\}$. For each match in *H_i_*, we denote the corresponding taxonomic classification as $T_{i}=\{p_{1},p_{2},...,p_{m}\}$, which represents the taxonomic affiliation of the genome that encodes the matching gene; and at taxonomic rank *t*, the labels (taxa) are $T_{i}^{t}=\{p_{1}^{t},p_{1}^{t},...,p_{m}^{t}\}$. We also denote the gene cluster each query sequence is assigned (matched) to as $C_{i}=\{c_{1},c_{2},...,c_{m}\}$. The top *N* matches of the *l^th^* gene against the reference database are used to weight different taxa (superscripted as *k*) at a specific rank (subscripted as *t*),$p_{t}^{k}$ (*N* is defined by the user; see also below), and the weight is:

$W_{l}(p_{t}^{k})=\sum_{\{j|p_{t}^{j}=p_{t}^{k}\}}^{N} \left[D_{t}^{c_{j}}(i_{j})w_{t}^{D}+M_{t}^{c_{j}}w_{t}^{M}\right]s_{j}$,

where $w_{t}^{D}$ and $w_{t}^{M}=1-w_{t}^{D}$ are the weights for *D* and *M* at taxonomic level *t,* respectively. To select the optimal $w_{t}^{M}$ and $w_{t}^{D}$, a grid search was carried out to maximize the algorithm’s performance as described in the main text. Therefore, the relative weight of $p_{t}^{k}$ for the *l^th^* gene, $L_{l}(p_{t}^{k})$ is normalized to the sum of weights over different *k* (*i.e.*, different taxonomic classifications):

$L_{l}(p_{t}^{k})=\frac{W_{l}(p_{t}^{k})}{\sum_{k} W_{l}(p_{t}^{k})}$.

And, the likelihood score of a specific taxon *k* at rank *t*, $p_{t}^{k}$, over the whole query sequence is:

$L(p_{t}^{k})=\frac{\sum_{l=1}^{m} L_{l}(p_{t}^{k})}{\sum_{k} \sum_{l=1}^{m} L_{l}(p_{t}^{k})}$.

**4. Mathematical definitions of sensitivity and specificity**

The taxonomic assignment of a “known” sequence by MyTaxa or another tool was denoted as “true prediction” (TP; predicted taxon matches the actual taxon), “wrong prediction” (WP; predicted taxon does not match the actual taxon), or “false negative” (FN; predicted as “unknown”); while the assignment of an “unknown” sequence was denoted either “false positive” (FP; predicted to match a specific taxon), or “true negative” (TN; predicted as “unknown”). Accordingly, the sensitivity of the algorithm was defined as:

$Sn=\frac{1}{N+M}\left(\sum_{i=1}^{N} \frac{n_{TP}^{i}}{n^{i}}+\sum_{i=1}^{M} \frac{n_{TN}^{i}}{m^{i}}\right)$ **,**

where *N* is the total number of taxa for the “known” sequences, and *M* is the total number of taxa for the “unknown” sequences. *n^i^* is the number of “known” sequences for each taxon, and *m^i^* is the number of “unknown” sequences for each taxon. $n_{TP}^{i}$ is the number of TP in the *i^th^* taxon and $n_{TN}^{i}$ is the umber of TN in the *i^th^* taxon. Similarly, the specificity of the algorithm was defined as:

$Sp=\frac{1}{N}\sum_{i=1}^{N} \frac{n_{TP}^{i}}{n^{i}}$.

**Supplementary tables**

**Table S1. The number of known and unknown taxa in draft genomes (*in-silico* query test dataset) compared to completed genomes (reference database).** Details on which specific genomes were used and denoted as “known” vs. “unknown” are provided in supplementary data accessible from http://enve-omics.ce.gatech.edu/MyTaxa.

| Rank | Number of known taxa; percentage (%) | Number of unknown taxa, percentage (%) |
| --- | --- | --- |
| Phylum | 20; 87.0% | 3; 13% |
| Genus | 143; 50.7% | 139; 49.3% |
| Species | 136; 18.5% | 600; 71.5% |

**Table S2. The number of known and unknown sequences at different taxonomic ranks in the *in-silico* query test datasets used for performance evaluation, and the percentage of sequences with at least one significant match in BLAT search.**

| Length (bp) | Phylum | | Genus | | Species | | Percent with significant match (%) |
| --- | --- | --- | --- | --- | --- | --- | --- |
|  | Known | Unknown | Known | Unknown | Known | Unknown |  |
| 100 | 954676 | 7168 | 814224 | 121620 | 527684 | 408160 | 32.7 |
| 500 | 493493 | 4234 | 429146 | 69981 | 290555 | 208572 | 38.5 |
| 800 | 494576 | 3909 | 430989 | 67496 | 287334 | 211151 | 41.2 |
| 1000 | 494186 | 3534 | 423663 | 73757 | 268881 | 228539 | 44.3 |
| 1500 | 296648 | 1536 | 252148 | 46036 | 148360 | 149824 | 47.8 |
| 2000 | 198286 | 1242 | 172622 | 26906 | 110584 | 88944 | 51.2 |

**Table S3. Detailed performance of classification tools on *in-silico* query test datasets.** This data underlie the results shown in figure 3.

|  | Phylum | | | | | Genus | | | | | Species | | | | |  |
| --- | --- | --- | --- | --- | --- | --- | --- | --- | --- | --- | --- | --- | --- | --- | --- | --- |
|  | TP^6^ | TN | WP | FP | FN | TP | TN | WP | FP | FN | TP | TN | WP | FP | FN |  |
| 100bp | 95.6 | 0 | 3.1 | 0.8 | 0.5 | 80 | 1.2 | 10.4 | 3.8 | 4.7 | 33.4 | 8.0 | 30.8 | 21.6 | 6.2 | MyTaxa |
|  | 78.4 | 0 | 20.8 | 0.7 | 0.2 | 69.7 | 0 | 26.1 | 4.0 | 0.2 | 34.5 | 0 | 41.2 | 24.0 | 0.2 | BH^2^ |
|  | 95.0 | 0 | 2.9 | 0.9 | 0.2 | 77.8 | 1.5 | 6.9 | 10.8 | 7.6 | 30.6 | 8.0 | 13.3 | 27.5 | 20.6 | LCA^3^ |
|  | 95.6 | 0.2 | 1.5 | 0.5 | 2.1 | 70.2 | 2.9 | 2.2 | 4.2 | 20.5 | 17.5 | 21.7 | 2.0 | 13.5 | 45.3 | MGR^4^ |
|  | 93.1 | 0 | 6.0 | 0.9 | 0 | 26.8 | 0 | 65.5 | 7.8 | 0 | 26.4 | 0 | 38.1 | 35.5 | 0 | MEGAN4 |
|  | 42.1 | 0.1 | 19.1 | 0.5 | 38.2 | 10.5 | 6.8 | 3.1 | 0.5 | 79.1 | 2.4 | 33.8 | 2.5 | 1.2 | 60.1 | PPS^5^ |
|  | 82.2 | 0 | 16.8 | 0.9 | 0 | 66.6 | 0 | 25.7 | 7.7 | 0 | 32.1 | 0 | 32.6 | 35.4 | 0 | NBC |
|  | 53.5 | 0 | 43.4 | 1.0 | 2.1 | 17.6 | 0 | 73.9 | 7.7 | 0.7 | N/A^1^ | | | | | RAIPhy |
| 500bp | 96.9 | 0.1 | 1.9 | 0.4 | 0.7 | 78.6 | 2.9 | 7.5 | 3.7 | 7.3 | 33.4 | 16.1 | 23.2 | 15.1 | 12.2 | MyTaxa |
|  | 87.4 | 0 | 11.8 | 0.4 | 0.4 | 75.9 | 0 | 17.7 | 5.9 | 0.3 | 32.9 | 0 | 38.7 | 28.0 | 0.3 | BH |
|  | 95.5 | 0 | 2.0 | 0.5 | 2.0 | 77.8 | 3.3 | 6.1 | 5.7 | 7.1 | 35.2 | 9.9 | 9.9 | 25.8 | 19.1 | LCA |
|  | 97.4 | 0.1 | 0.6 | 0.6 | 0 | 71.0 | 3.4 | 1.7 | 0.7 | 16.6 | 15.8 | 24.7 | 1.6 | 18.2 | 39.8 | MGR |
|  | 93.8 | 0 | 5.6 | 0.4 | 1.5 | 25.9 | 0 | 65.1 | 9.0 | 0 | 27.5 | 0 | 36.8 | 35.7 | 0 | MEGAN |
|  | 44.7 | 0.4 | 17.4 | 0.3 | 37.1 | 12.3 | 7.9 | 2.9 | 0.4 | 76.4 | 2.5 | 34.6 | 2.2 | 0.8 | 59.9 | PPS |
|  | 87.5 | 0 | 11.9 | 0.6 | 0 | 73.8 | 0 | 17.2 | 9.0 | 0 | 36.9 | 0 | 27.4 | 35.7 | 0 | NBC |
|  | 82.1 | 0 | 15.6 | 0.5 | 1.6 | 48.8 | 0 | 41.7 | 8.9 | 0.5 | N/A | | | | | RAIPhy |
| 800bo | 97.6 | 0 | 1.4 | 0.3 | 0.6 | 79.1 | 3.0 | 7.2 | 3.6 | 7.1 | 35.4 | 17.2 | 20.0 | 14.9 | 12.6 | MyTaxa |
|  | 86.0 | 0 | 13.2 | 0.3 | 0.4 | 74.4 | 0 | 19.4 | 5.7 | 4.2 | 32.9 | 0 | 38.5 | 28.2 | 0.3 | BH |
|  | 95.9 | 0 | 1.4 | 0.4 | 2.3 | 76.1 | 3.6 | 5.3 | 5.3 | 9.6 | 34.2 | 12.0 | 7.6 | 24.8 | 21.4 | LCA |
|  | 98.0 | 0.1 | 0.4 | 0.3 | 1.2 | 71.4 | 3.5 | 1.2 | 9.3 | 14.6 | 16.4 | 26.2 | 1.2 | 22.2 | 33.9 | MGR |
|  | 94.3 | 0 | 5.3 | 0.4 | 0 | 26.2 | 0 | 64.8 | 9.0 | 0 | 26.6 | 0 | 36.6 | 36.8 | 0 | MEGAN |
|  | 48.3 | 0.2 | 12.6 | 0.2 | 38.7 | 13.5 | 8.5 | 2.0 | 0.4 | 75.6 | 2.5 | 35.8 | 2.2 | 1.0 | 58.5 | PPS |
|  | 85.9 | 0 | 13.7 | 0.4 | 0 | 70.7 | 0 | 20.3 | 9.0 | 0 | 34.3 | 0 | 29.0 | 36.8 | 0 | NBC |
|  | 87.4 | 0 | 10.8 | 0.4 |  | 56.5 | 0.1 | 34.2 | 8.9 | 0.3 | N/A | | | | | RAIPhy |
| 1000bp | 97.7 | 0 | 1.3 | 0.3 | 0.6 | 78.1 | 3.7 | 7.0 | 4.0 | 7.2 | 34.3 | 19.8 | 17.7 | 15.7 | 12.4 | MyTaxa |
|  | 88.7 | 0 | 10.6 | 0.3 | 0.5 | 75.5 | 0 | 19.0 | 7.7 | 0.6 | 35.5 | 0 | 31.9 | 32.2 | 0.4 | BH |
|  | 95.6 | 0 | 1.3 | 0.3 | 1.2 | 73.9 | 4.7 | 4.9 | 5.6 | 10.8 | 31.9 | 15.0 | 6.3 | 25.4 | 21.4 | LCA |
|  | 98.0 | 0.1 | 0.4 | 0.3 | 1.2 | 71.4 | 3.5 | 1.2 | 9.3 | 14.6 | 16.4 | 26.2 | 1.2 | 22.2 | 33.9 | MGR |
|  | 94.1 | 0 | 5.5 | 0.3 | 0 | 27.5 | 0 | 62.2 | 10.3 | 0 | 25.5 | 0 | 34.0 | 40.5 | 0 | MEGAN |
|  | 50.0 | 0.2 | 11.1 | 0.1 | 38.5 | 14.6 | 9.8 | 1.8 | 0.5 | 73.2 | 2.8 | 39.4 | 2.3 | 1.1 | 54.4 | PPS |
|  | 88.2 | 0 | 11.2 | 0.6 | 0 | 67.9 | 0 | 21.8 | 10.3 | 0 | 35.6 | 0 | 28.6 | 35.8 | 0 | NBC |
|  | 88.9 | 0 | 9.5 | 0.3 | 1.3 | 58.6 | 0 | 30.8 | 10.3 | 0.3 | N/A | | | | | RAIPhy |
| 1500bp | 98.0 | 0 | 1.2 | 0.2 | 0.6 | 77.2 | 4.3 | 6.2 | 4.6 | 7.7 | 32.5 | 23.7 | 14.0 | 17.4 | 12.3 | MyTaxa |
|  | 86.4 | 0 | 10.6 | 0.3 | 0.5 | 72.3 | 0 | 19.0 | 7.7 | 0.6 | 33.4 | 0 | 29.8 | 36.3 | 0.4 | BH |
|  | 95.6 | 0 | 1.3 | 2.9 | 2.8 | 74.3 | 4.3 | 4.7 | 6.3 | 10.4 | 30.7 | 15.1 | 5.5 | 29.3 | 19.4 | LCA |
|  | 98.0 | 0.1 | 0.4 | 2.9 | 1.2 | 71.0 | 3.8 | 1.1 | 10.6 | 0 | 15.3 | 30.2 | 0.9 | 25.5 | 28.2 | MGR |
|  | 94.1 | 0 | 5.5 | 0.3 | 0 | 27.6 | 0 | 61.7 | 10.7 | 0 | 24.2 | 0 | 31.4 | 44.4 | 0 | MEGAN |
|  | 50.0 | 0.2 | 11.1 | 0.1 | 38.5 | 18.7 | 10.1 | 1.6 | 0.6 | 69.0 | 3.4 | 42.7 | 2.8 | 1.7 | 49.4 | PPS |
|  | 88.3 | 0 | 11.3 | 0.4 | 0 | 67.3 | 0 | 22.0 | 10.7 | 0 | 31.7 | 0 | 23.9 | 44.4 | 0 | NBC |
|  | 91.9 | 0 | 6.8 | 0.2 | 1.0 | 64.2 | 0 | 25.0 | 10.6 | 0.2 | N/A | | | | | RAIPhy |
| 2000bp | 98.1 | 0 | 1.0 | 0.2 | 0.6 | 80.6 | 3.7 | 4.8 | 4.2 | 6.8 | 34.3 | 21.7 | 15.0 | 15.0 | 14.0 | MyTaxa |
|  | 82.8 | 0 | 16.4 | 0.2 | 0.6 | 71.7 | 0 | 21.1 | 6.6 | 0.6 | 34.1 | 0 | 34.4 | 31.0 | 0.5 | BH |
|  | 95.8 | 0 | 1.0 | 0.3 | 2.9 | 77.2 | 4.0 | 3.6 | 5.4 | 9.8 | 30.9 | 14.8 | 5.1 | 24.7 | 24.4 | LCA |
|  | 97.8 | 0 | 0.5 | 0.3 | 1.3 | 71.0 | 4.3 | 1.5 | 10.0 | 13.1 | 16.2 | 28.9 | 1.7 | 25.1 | 28.1 | MGR |
|  | 94.3 | 0 | 5.4 | 0.3 | 0 | 26.6 | 0 | 64.1 | 9.4 | 0 | 28.8 | 0 | 31.7 | 40.0 | 0 | MEGAN |
|  | 61.2 | 0.2 | 7.1 | 0.1 | 31.3 | 25.1 | 8.8 | 1.4 | 0.6 | 64.1 | 4.6 | 37.7 | 4.2 | 1.9 | 51.7 | PPS |
|  | 88.3 | 0 | 11.3 | 0.4 | 0 | 68.2 | 0 | 21.5 | 10.3 | 0 | 37.0 | 0 | 21.2 | 41.8 | 0 | NBC |
|  | 93.1 | 0 | 5.6 | 0.3 | 1.0 | 70.4 | 0.1 | 20.0 | 9.3 | 0.2 | N/A | | | | | RAIPhy |

* The numbers represent percentages of the total;

^1^ N/A, not available, RAIPhy does not provide species level prediction;

^2^ BH, best hit; ^3^ LCA, lowest common ancestor; ^4^ MGR, MG-RAST ([39](#_ENREF_1)); ^5^ PPS, PhyloPythiaS ([40](#_ENREF_2));

^6^ TP, true prediction; TN, true negative; WP, wrong prediction; FP, false positive; FN, false negative.

**Table S4. The datasets used for evaluating the impact of the degree of novelty of the query sequence on MyTaxa’s prediction accuracy.** Novelty of a dataset was defined as the percentage of query sequences originated from taxa that are “unknown” to reference database used to calculate gene cluster weights.

|  | **Low novelty** | | **Medium novelty** | | **High novelty** | |
| --- | --- | --- | --- | --- | --- | --- |
| Rank | **Total sequences** | **Novel sequence range (%)** | **Total sequences** | **Novel sequence range (%)** | **Total sequences** | **Novel sequence**  **range (%)** |
| **Species** | 5,964 | 7%-16% | 5,335 | 18%-41% | 6,000 | 36%-54% |
| **Genus** | 6,000 | 5%-15% | 6,000 | 12%-31% | 6,000 | 28%-45% |
| **Phylum** | 6,000 | 2%-15% | 6,000 | 8%-22% | 6,000 | 14%-33% |

**Table S5. Database search time using different search tools.** Gene sequences were predicted on the *in-silico* generated query test datasets and subsequently searched against NCBI all complete genomes (release 196) using BLAST, BLAT, and Usearch with default settings. The searches were carried out on a 2.4 GHz Xeon processor with 8 Gbp RAM Linux box running Red Hat Enterprise 6.3, using a single thread.

|  | Processing speed (number of sequences per second) | | | | | |
| --- | --- | --- | --- | --- | --- | --- |
|  | 100bp | 500bp | 800bp | 1000bp | 1500bp | 2000bp |
| BLASTP^a^ | 0.21 | 0.17 | 0.14 | 0.11 | 0.09 | 0.07 |
| BLAT^b^ | 8.31 | 8.21 | 8.03 | 7.81 | 7.74 | 7.51 |
| USearch^c^ | 12.31 | 11.21 | 10.52 | 9.43 | 8.97 | 8.57 |

**Supplementary figures**

**Figure S1. The impact of the number of matches used in the analysis on the classification accuracy of MyTaxa.** The impact of the number of top matches analyzed, *N*, on MyTaxa accuracy was evaluated, for query sequences of varied length (figure key) and each taxonomic rank (labels on top). *N* = 5 typically performed the best, both in terms of accuracy (y-axes) as well as computational demand (data not shown).

**Figure S2. The impact of the likelihood score cutoff on the classification accuracy of MyTaxa.** The impact of different likelihood cut-offs in the maximum likelihood analysis was evaluated using a similar approach as that shown in Figure S1. Scores of 0.5 typically perform the best (y-axes) and were efficient in terms of computational demand.

**Figure S3. Composition of *in-silico* generated test dataset at phylum level.** Each ring represent a dataset of different read length (from inside outwards): 100bp, 500bp, 800bp, 1000bp, 1500bp, and 2000bp.

**Figure S4. Accuracy of MyTaxa in comparison with other homology-based methods at the species level.** The accuracy (TP+TN) of different methods (figure key; y-axis) as a function of the length of the query sequence (x axis) is shown. Note that MyTaxa correctly assigns at least 3%, and up to 32%, more sequences than any other method, depending on the length of the query sequences. Figure S4 is similar to Figure 3 but represents the sum of the true positives (TP) and true negatives (TN) results, zooming in at the species level.

**
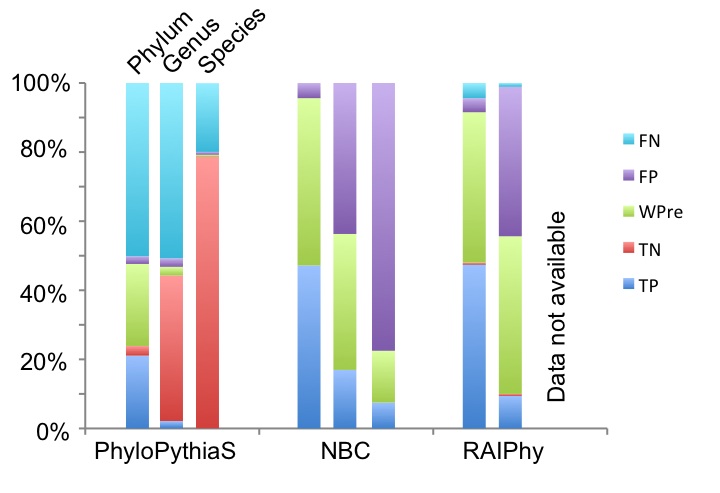
**

**Figure S5. Performance of composition-based methods on sequences that did not have significant matches in the reference database.** Composition-based methods typically classify all input sequences, including those without significant matches with the reference database. However, significant lower accuracies were observed on these sequences compared to those with significant matches, e.g., NBC’s accuracy was 16.9% versus 36.9% at the genus level, respectively; presumably due to the fact that sequences with no significant matches (ranging from 48.8% to 67.3% in our *in-silico* query datasets) tend to represent taxon-specific genes, which are often the product of horizontal gene transfer ([41](#_ENREF_3)). The types of predictions are color-coded in the same way as in Figure 3.

**Figure S6. Run-time analysis of MyTaxa in comparison with other homology-based tools.** All tests were performed on a single 2.4GHz Xeon quad core CPU with Red Hat Enterprise 6.3, using 1000bp long reads, and the processing time was measured as the number of entries processed per second (*y-axis*), not counting the similarity search time.

**Figure S7. Classifying of taxa into low, medium, and high abundance based on the number of genomes available in the reference database.** The taxa (species, genera or phyla) were ranked based on the number of available genomes in each taxon (graph) and taxa were denoted as low, medium and high abundance based on the number of genomes (see figure key). The cut-off chosen for each abundance category were selected to represent the different phases of the abundance curve.

**Figure S8. Prediction accuracy of different tools as an effect of the degree of novelty of the input dataset.**The prediction accuracy (*y*-axis) at genus level of each tool on both low novelty datasets (solid line) and high novelty datasets (dashed line) was measured at different input sequence lengths (*x*-axis). Prediction accuracy is defined as the sum of the percentage of true positives (TP) and true negatives (TN; for detailed definitions, see Materials and Methods).

**Figure S9. Genera relative abundance in a biogas-producing metagenome based on different taxonomic classifiers.** The relative abundance (*x* axis) of each genus (*y* axis) was based on the taxonomic classification of 616,072 Roche 454 FLX shotgun reads by each classifier (figure key). PhyloPythiaS was not included in the analysis due to the low number of reads assigned to the genus level (<5% of the total reads).


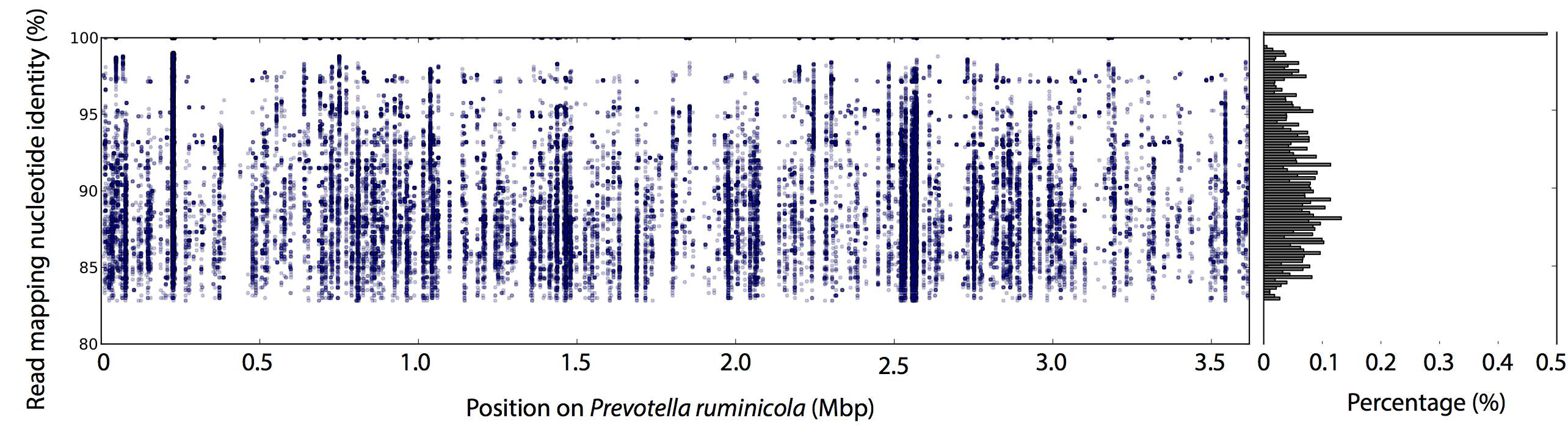


**Figure S10. Population structure of *Prevotella* in the HMP metagenome.** The graph shows the read recruitment plot against the *Prevotella* strain 23 (NC_01433) reference genome sequence, i.e., each dot represents a read that matched the reference sequence with high nucleotide identity (*y*-axis) plotted against the location of the match on the reference (*x*-axis). Right panel shows the coverage (i.e., the percent of the total reads that matched the reference) at each unit of nucleotide identity. Note that only a minor fraction of the total reads showed high nucleotide identity to the reference (i.e., 95% or higher). Most reads showed 85-90% nucleotide identity to the reference, indicating that they represent closely related but probably distinct species compared to the reference genome accordingly to the most frequently used taxonomic standards ([42](#_ENREF_4)).

**Supplementary references**

39. Glass, E.M., Wilkening, J., Wilke, A., Antonopoulos, D. and Meyer, F. (2010) Using the metagenomics RAST server (MG-RAST) for analyzing shotgun metagenomes. *Cold Spring Harbor protocols*, **2010**, pdb prot5368.

40. Patil, K.R., Haider, P., Pope, P.B., Turnbaugh, P.J., Morrison, M., Scheffer, T. and McHardy, A.C. (2011) Taxonomic metagenome sequence assignment with structured output models. *Nature methods*, **8**, 191-192.

41. Daubin, V. and Ochman, H. (2004) Bacterial genomes as new gene homes: the genealogy of ORFans in E. coli. *Genome Res*, **14**, 1036-1042.

42. Goris, J., Konstantinidis, K.T., Klappenbach, J.A., Coenye, T., Vandamme, P. and Tiedje, J.M. (2007) DNA-DNA hybridization values and their relationship to whole-genome sequence similarities. *International journal of systematic and evolutionary microbiology*, **57**, 81-91.
